# The Optimized γ-Globin Lentiviral Vector GGHI-mB-3D Leads to Nearly Therapeutic HbF Levels In Vitro in CD34^+^ Cells from Sickle Cell Disease Patients

**DOI:** 10.3390/v14122716

**Published:** 2022-12-05

**Authors:** Ekati Drakopoulou, Maria Georgomanoli, Carsten W. Lederer, Fottes Panetsos, Marina Kleanthous, Ersi Voskaridou, Dimitrios Valakos, Eleni Papanikolaou, Nicholas P. Anagnou

**Affiliations:** 1 Laboratory of Cell and Gene Therapy, Centre of Basic Research, Biomedical Research Foundation of the Academy of Athens (BRFAA), 11527 Athens, Greece; 2 Laboratory of Biology, School of Medicine, National and Kapodistrian University of Athens, 11527 Athens, Greece; 3 The Molecular Genetics Thalassemia Department, The Cyprus Institute of Neurology and Genetics, 2371 Nicosia, Cyprus; 4 Bioiatriki SA Health Group Company, 11526 Athens, Greece; 5 Thalassemia and Sickle Cell Disease Centre, Laiko General Hospital, 11527 Athens, Greece; 6 Laboratory of Molecular Biology, Centre of Basic Research, Biomedical Research Foundation of the Academy of Athens (BRFAA), 11527 Athens, Greece

**Keywords:** sickle cell disease, thalassemia, globin gene therapy, γ-globin lentiviral vector, HbF, HPFH enhancers, -117 HPFH type, CD34^+^ hematopoietic stem cells

## Abstract

We have previously demonstrated that both the original γ-globin lentiviral vector (LV) GGHI and the optimized GGHI-mB-3D LV, carrying the novel regulatory elements of the 3D HPFH-1 enhancer and the 3’ β-globin UTR, can significantly increase HbF production in thalassemic CD34^+^ cells and ameliorate the disease phenotype in vitro. In the present study, we investigated whether the GGHI-mB-3D vector can also exhibit an equally therapeutic effect, following the transduction of sickle cell disease (SCD) CD34^+^ cells at MOI 100, leading to HbF increase coupled with HbS decrease, and thus, to phenotype improvement in vitro. We show that GGHI-mB-3D LV can lead to high and potentially therapeutic HbF levels, reaching a mean 2-fold increase to a mean value of VCN/cell of 1.0 and a mean transduction efficiency of 55%. Furthermore, this increase was accompanied by a significant 1.6-fold HbS decrease, a beneficial therapeutic feature for SCD. In summary, our data demonstrate the efficacy of the optimized γ-globin lentiviral vector to improve the SCD phenotype in vitro, and highlights its potential use in future clinical SCD trials.

## 1. Introduction

Sickle cell disease (SCD) is a monogenic disorder caused by a single amino acid substitution in the β-globin gene (glu(E)6Val(A); GAG → GTG; rs334), which results in a multi-organic disease phenotype [1,2,3]. It is characterized by the polymerization of deoxygenated sickle hemoglobin (HbS; a_2_β^S^_2_), leading to sickle-shaped red blood cells, and thus, to vaso-occlusion and hemolytic anemia, which are accompanied by organ damage and painful crisis [4,5]. Patients with SCD have a shortened life span, and suffer from severe clinical manifestations, such as acute painful episodes, leg ulcers [6,7], osteonecrosis, chest pain, priapism, stroke, silent cerebral infarction, systemic high blood pressure, and, less frequently, from sickle vasculopathy [8,9].

The beneficial effect of fetal HbF on SCD outcome becomes apparent as early as 6 months after birth, where high levels of HbF lead to asymptomatic disease. SCD patients who continue to produce increased (>20%) γ-globin levels in adult life exhibit a less severe phenotype [4,10], primarily due to decreased HbS polymerization [8,10]. Therapies utilizing HbF inducers, such as 5-azacytidine [11,12] and hydroxyurea (HU) [13,14,15], or blood transfusion, combined with iron chelation, offer relief to SCD patients [16], as they alleviate disease symptoms.

Alternative therapeutic approaches, such as gene therapy, can also be curative in SCD, as reported by Ribeil et al. in a clinical trial where a patient with severe SCD underwent gene therapy and exhibited a high proportion of anti-sickling hemoglobin post transplant, which accounted for 45% of the total hemoglobin production [17]. Gene therapy studies utilizing LVs containing either an anti-sickling β^T87Q^-globin or γ-globin transgenes was shown to ameliorate the SCD clinical manifestation either in vivo in SCD mouse models [18,19,20] or in vitro using CD34^+^ cells from SCD patients [21,22,23,24]. Additionally, the incorporation of small hairpin RNA (shRNA) in therapeutic globin LVs, either for simultaneous reduction in β^S^ transcripts [25] or for down-regulation of the BCL11A expression [26], can lead to SCD phenotype correction. Furthermore, the role of many miRNAs that bind to transcription factors such as BCL11A, GATA-1, KLF-1, and MYB, is crucial in reactivating the γ-globin gene expression [27,28]. To this end, Sankaran et al. showed that there is a delay in silencing and persistence of fetal hemoglobin coupled with an elevation in embryonic hemoglobin in newborns, attributed to miR-15a and miR-16-1 directly targeting MYB [29,30]. Finally, following the gene editing approach, Magis et al. recently managed to successfully correct the sickle cell mutation in more than 30% of the long-term engrafting hHSCs, using a high-fidelity Cas9 nucleoprotein (RNP) and a single-stranded oligonucleotide donor [31]. Corrected erythroblasts showed a clear dominance of the corrected allele over null β-thalassemia alleles produced by non-homologous end joining (NHEJ), demonstrating a marked survival advantage in vivo [31]. 

We have previously demonstrated that the original LCR-free, self-inactivating (SIN), insulated γ-globin lentiviral vector GGHI [32], containing the HPFH-2 enhancer element (shown to lead to elevated HbF levels [33,34]), the -117 activating HPFH mutation of the ^A^γ gene promoter, and the HS-40 enhancer from the α-globin locus [35], led to in vitro correction of the thalassemic phenotype [32]. The use of the HS-40 enhancer element instead of the LCR core elements of the β-globin locus was shown to efficiently enhance the expression of the ^A^γ-globin gene, and was also associated with high functional titers and genomic stability [32,34], features that the globin vectors containing several LCR elements usually lack, while they are prone to genomic rearrangements and trans-activation of cancer-related genes [36,37]. Furthermore, in the novel, improved GGHI-mB-3D vector [38], we have incorporated the 3D enhancer element of the naturally occurring HPFH-1 deletion [39,40], along with the β-globin gene 3’ UTR [18], and pseudotyped it with the alternative BaEVRless envelope glycoprotein [41,42], which resulted in high and stable HbF expression at low multiplicities of infections (MOIs) in thalassemic CD34^+^ cells. 

Based on the previous successful performance of both vectors in thalassemic CD34^+^ cells, in this study, we evaluated their ability to correct the SCD phenotype in vitro. To this end, CD34^+^ cells from non-mobilized peripheral blood of SCD patients were transduced with the GGHI or GGHI-mB-3D vectors, pseudotyped with the VSVG envelope glycoprotein. We show that transduction with the optimized GGHI-mB-3D vector leads to a significant increase in the ^A^γ/α ratio and HbF, along with a concomitant decrease in HbS in our patient cohorts, thus demonstrating an increased potential of improving, as well, the SCD phenotype in vitro compared to GGHI. The above effects were achieved at a transduction efficiency of 55% and a clinically relevant mean vector copy number (VCN)/cell of 1.0.

## 2. Materials and Methods

### 2.1. Virus Production and Titration

GGHI and GGHI-mB-3D LVs were produced by transient co-transfection of HEK 293T cells using a third-generation lentiviral system [43,44] comprising the following plasmids: vector (GGHI or GGHI-mB-3D), packaging plasmids (pMDLg/pRRE and pRSV-Rev), and envelope plasmids encoding the vesicular stomatitis virus glycoprotein VSVG (pMD2.VSVG). All accessory plasmids were generously donated by Dr Luigi Naldini (additional information on these plasmids can be also found at www.addgene.com) (accessed on 1 December 2022). Virus-containing supernatant was collected at 48 h and 72 h following transfection, as previously described by Papanikolaou et al. [45]. Viral titers were determined by transducing 5 × 10^5^ mouse erythroleukemia (MEL-585) cells, using serial dilutions of concentrated virus, followed by their induction to erythroid differentiation using 10 μM hemin (Sigma-Aldrich, St. Louis, MO, USA) and 3 mM of HMBA (N,N′-hexamethylene bisacetamide, Sigma-Aldrich, St. Louis, MO, USA). HbF expression was measured by fluorescence-activated cell sorter scanner (FACS) using an anti-HbF FITC conjugated monoclonal antibody (BD Biosciences, Franklin Lakes, NJ, USA).

### 2.2. Human CD34^+^ Stem Cell Isolation and Transduction: Sample Collection and Processing

We used CD34^+^ hematopoietic stem cells isolated from five homozygote patients for SCD (β^S^β^S^) and five compound heterozygotes (β^S^β^+^) for SCD and β-thalassemia. All samples were obtained from non-mobilized peripheral blood and harvested from volunteer donors, using protocols approved by the Institutional Review Board of the Laiko General Hospital of Athens, in accordance with the Helsinki declaration of 1975. Informed consent was obtained from all subjects involved in the study. All samples were obtained shortly before the next scheduled blood transfusion. CD34^+^ cells were isolated from mononuclear cells using an EasySep™ Human CD34 Positive Selection Kit (Stem Cell Technologies, Cambridge, UK), according to the manufacturer’s instructions. Samples obtained were >90% enriched with CD34^+^ cells, and were further cultured for up to 21 days in erythroid liquid cultures, as described previously [32,38]. Analyses of liquid cultures included high-performance liquid chromatography (HPLC) and flow cytometry for the assessment of the ^A^γ/α ratio, and HbF and HbS production, and flow cytometry for the assessment of apoptosis using the Annexin V/7-AAD detection kit (BioLegend, San Diego, CA, USA) on day 20–21. At day 20–21, 10^5^ cells were removed from culture and used for RNA isolation, as described below. A schematic representation of the experimental procedure is shown in Figure S1A.

### 2.3. Reversed-Phase High-Performance Liquid Chromatography (RP-HPLC) Analysis for Globin Chain ^A^γ/α Ratio Quantitation

Reversed-phase high-performance chromatography was performed with slight modification of published methods [46]. In brief, cell material was pelleted at 2000 RCF for 10 min and resuspended in H_2_O supplemented with 5 mM dithiothreitol at a concentration of 20,000 cells/μL. Following two freeze–thaw cycles and a 10 min centrifugation at 21,000 RCF at 4 °C, the supernatant was transferred to 100-μL HPLC microvials (Altmann Analytik, Munich, Germany), and 10–30 μL were injected per run. Analyses were performed on a Prominence HPLC machine with SPD-M20A diode array detector and LC-20AD pump (Shimadzu, Kyoto, Japan), in combination with a Jupiter 5 μm C18 4.6 mm column and corresponding guard columns (Phenomenex, Torrance, CA, USA) using an increasing gradient of phase B, i.e., 0.1% trifluoroacetic acid in acetonitrile (Sigma-Aldrich, St. Louis, MO, USA), against phase A, i.e., 0.1% trifluoroacetic acid (Sigma-Aldrich, St. Louis, MO, USA) and 6.4 mM sodium hydroxide.

### 2.4. Hemoglobin Electrophoresis and Cation Exchange HPLC (CE-HPLC) for HbF and HbS Quantitation

HbF and HbS quantification in β^S^β^+^ samples was performed using the Hydragel-Hemoglobin(e) K20 kit (SEBIA, Lisses, France) according to the manufacturer’s instructions. Briefly, 10^6^–10^7^ cells were harvested from liquid cultures on day 20–21 and hemolyzed using 10–20 μL of hemolyzing solution; 10 μL of the hemolysate was loaded onto a Hydragel K20 application carrier according to the manufacturer’s instructions. HbF and HbS quantification was performed using QuantityOne software (Bio-Rad, CA, USA) and densiometric analysis. HbF and HbS quantification in β^S^β^S^ samples was performed by CE-HPLC as described previously by Papanikolaou et.al [32].

### 2.5. RNA Analysis and Measurement of γ-Globin Transcript Levels Using Quantitative Real-Time PCR

Quantitation of γ-globin transcripts was carried out employing quantitative real-time PCR (qPCR) using SYBR^TM^ Green mix (Kapa Biosystems, Wilmington, MA, USA), and performed on a CFX Connect^TM^ Real-Time System (Bio-Rad, Hercules, CA, USA). For the quantification of γ-globin production from erythroid cultures, total RNA was isolated from both mock-transduced and transduced CD34^+^ cells maintained at day 20–21, using the RNeasy kit (Qiagen, Germantown, MD, USA) and according to the manufacturer’s instructions. A quantity of 5–500 ng of total RNA was reverse transcribed to cDNA using the Superscript First-Strand Synthesis System for RT-PCR (Invitrogen, Carlsbad, CA, USA), and 20 ng of c-DNA was subjected to qPCR analysis. Production of γ-globin was measured using the following primers: gamma F: 5’-GCCATAAAGCACCTGGATGA-3’, and gamma R: 5’-GATTGCCAAAACGGTCACC-3’. The human α-globin gene was used as a reference gene using the following primers: alpha-globin F: 5’-CACGCTGGCGAGTATGGTG-3’ and alpha-globin R: 5’-TTAACCCTGGGCAGAGCCGT-3’. Fold increase in γ-globin mRNA in transduced and mock-transduced cell populations was calculated using the ΔΔCt [47] method. Measurements of human γ-globin mRNA levels were performed in duplicate for each sample. The amplification was carried out according to the conditions suggested by the manufacturer.

### 2.6. Flow Cytometry

Transduced MEL-585 cells were induced towards erythroid phenotype and were then processed for flow cytometry analysis as previously described [38], using an anti-HbF FITC-conjugated monoclonal antibody (BD Pharmingen, Franklin Lakes, NJ, USA). Similarly, CD34^+^ cells from erythroid cultures were stained with both anti-HbF FITC and anti-Glycophorin A-PE conjugated monoclonal antibodies (BD Pharmingen, Franklin Lakes, NJ, USA) and subjected to flow cytometry analysis. 

Apoptotic assays were performed on cells derived from erythroid cultures on day 20–21, employing FITC using the Annexin V/7-AAD detection kit with 7-AAD (BioLegend, San Diego, CA, USA) according to the manufacturer’s instructions. Briefly, cells were washed twice with cold PBS, containing 1% FBS, and resuspended in Annexin-V Binding buffer at a concentration of 0.25–1 × 10^7^ cells/mL. Cells were then incubated for 15 min at room temperature in the dark, and analyzed by flow cytometry within an hour. 

All samples were analyzed in a Cytomics FC 500 (CXP) Series Flow Cytometry System (Beckman Coulter, Nyon, Switzerland). Flow cytometry analysis was performed using FlowJo 10.8.1 analysis software.

### 2.7. Determination of Vector Copy Number and Transduction Efficiency

In order to assess the vector transduction efficiency, 10–20 burst-forming units (BFUe) per patient were subjected to semi-quantitative PCR analysis using the primers gamma F/R described above. DNA was isolated with the QIAamp DNA Micro Kit (Qiagen, MD, USA) according to the manufacturer’s instructions. Vector transfer efficiency in CD34^+^ cells was determined by assessing the proportion of BFUe colonies that tested positive for vector sequences. Those tested positive for vector-specific sequences were further subjected to absolute quantitation with qPCR analysis for VCN determination, using the primers gamma F/R and the hRNAseP primers, as previously described [38]. Analysis of each sample was performed in duplicate, using SYBR^TM^ Green mix (Kapa Biosystems, Wilmington, MA, USA) and according to manufacturer’s instructions.

### 2.8. Statistical Analysis

Repeated-measures ANOVA, followed by post hoc Tukey tests, were used to detect statistically significant differences among different treatments, and paired two-tailed Student’s *t*-tests for pairwise comparisons, unless stated otherwise. Pearson’s *r* correlation coefficient was applied for correlation between values. Statistical analyses were performed using Prism 9.3.1, IBM SPSS 28, and R 4.0.0 software, while graph creation was conducted with Prism 9.3.1 software.

## 3. Results

### 3.1. GGHI and GGHI-mB-3D LVs Exhibit High Titers

Both GGHI and GGHI-mB-3D LVs exhibited high mean titers; specifically, 1.63 × 10^8^ TU/mL (*n* = 6) and 1.56 × 10^8^ TU/mL (*n* = 5), respectively (Figure S1B). No statistical difference was observed between the mean values of GGHI and GGHI-mB-3D LVs titers (*p* = 0.902, unpaired two-tailed *t*-test).

### 3.2. Increased ^A^γ/α Chain Ratio in SCD CD34^+^ Cells following Transduction with GGHI-mB-3D Lentiviral Vector

Non-mobilized peripheral blood CD34^+^ cells from ten SCD patients were isolated and processed. Five were homozygotes for the sickle cell disease mutation and five were compound heterozygotes for SCD and β-thalassemia (β^S^β^+^). All patients had four intact α-globin genes. Typical yields from an initial volume of 20 mL of peripheral blood ranged from 2 × 10^5^ to 10^6^ CD34^+^ cells. All patients were receiving HU, and therefore, initial HbF levels were high, reaching a mean percentage of 68.3 ± 14.2% in erythroid cultures, as detected by flow cytometry. Baseline levels of HbF and HbS prior to HU treatment are shown in Figure S2A,B (upper panel).

**Figure 1:**
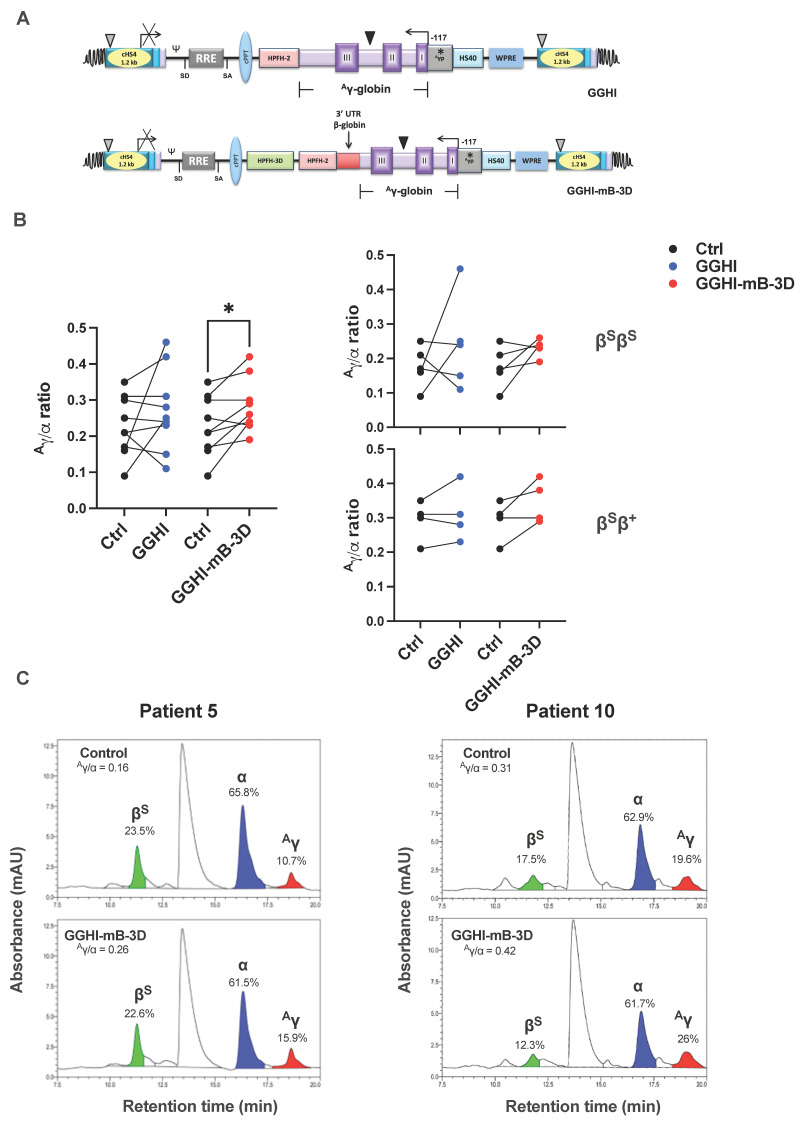
In vitro assessment of γ-globin lentiviral vectors (LVs) in SCD CD34^+^ cells: (**A**) The structure and the individual regulatory elements of the GGHI and GGHI-mB-3D LVs shown as proviral elements. The grey triangle represents the 398 bp deletion in the cHS4, the black triangle shows the 713 bp in the ^A^γ cassette, and the asterisk depicts the -117 point mutation in the ^A^γ promoter. (**B**) Before-and-after plot showing ^A^γ/α globin chain ratios obtained with RP-HPLC across patients, prior and post transduction, with GGHI (*p* = 0.297, *n* = 9, paired two-tailed *t*-test) and GGHI-mB-3D (*p* = 0.0189, *n* = 9, paired two-tailed *t*-test) LVs (left panel). Before-and-after plots showing ^A^γ/α globin chain ratios in the β^S^β^S^ and β^S^β^+^ patient cohort, prior and post transduction, with GGHI and GGHI-mB-3D LVs (right panel). For β^S^β^S^ patient cohort GGHI (*p* = 0. 41, *n* = 5, paired two-tailed *t*-test) and GGHI-mB-3D (*p* = 0.14, *n* = 5, paired two-tailed *t*-test), and for β^S^β^+^ patient cohort GGHI (*p* = 0.45, *n* = 4, paired two-tailed *t*-test) and GGHI-mB-3D *(p =* 0.12, *n* = 4, paired two-tailed *t*-test). (**C**) RP-HPLC chromatograms from Patient 5 (left panel) and Patient 10 (right panel). Percentages shown are relative to the sum of the highlighted peaks. Each dot corresponds to each patient. * *p* ≤ 0.05.

We initially asked whether both GGHI/VSVG and GGHI-mB-3D/VSVG γ-globin vectors (Figure 1A), previously tested on CD34^+^ cells from thalassemia patients [32,38], could increase the ^A^γ/α ratio in SCD patients as well. To this end, we isolated CD34^+^ cells from SCD donors and cultured them under serum-free conditions for 18 h, dividing them into three cell aliquots; two subpopulations were transduced with each type of γ-globin vectors for 24 h, while the third was mock-transduced and served as a control. In both cases, and since our vectors were both pseudotyped with the conventional VSVG envelope glycoprotein, the MOI used was 100, as described by Papanikolaou et al. [32]. Next day, the cells were washed twice in PBS and resuspended in erythroid medium, as described in Section 2.

RP-HPLC analysis demonstrated that GGHI-mB-3D-transduced cells led to a highly significant increase in mean ^A^γ/α chain ratio (*p* = 0.02, *n* = 9) (Figure 1B, left panel); specifically, from 0.23 ± 0.08 observed in the control to 0.28 ± 0.08 (Table 1), with a mean difference of 0.056 ± 0.057 (Figure S3A). On the contrary, and under the aforementioned conditions, GGHI LV failed to do so, leading to a mean ^A^γ/α ratio of 0.27 ± 0.11 (*p* = 0.30, *n* = 9), as presented in Table 1. However, no statistical difference was observed between GGHI and GGHI-mB-3D LVs (*p* = 0.74, *n* = 9). 

When patients were divided according to genotype, transduction of β^S^β^S^ and β^S^β^+^ cohorts with both GGHI and GGHI-mB-3D LVs showed a trend, but not a statistically significant increase. Specifically, as shown in Figure 1B (right panel) and Table 2, transduction with the GGHI-mB-3D lentiviral vector led to a ^A^γ/α ratio increase from 0.18 ± 0.06, observed in the mock-transduced cells, to 0.23 ± 0.03 (*p* = 0.14, *n* = 5), while the corresponding value post transduction with GGHI was 0.24 ± 0.14 (*p* = 0.41, *n* = 5). Mean ^A^γ/α ratio achieved by GGHI and GGHI-mB-3D LVs did not test significantly different (*p* = 0.86, *n* = 5). In the case of the compound heterozygote β^S^β^+^ patient cohort, the majority of patients showed an increase in the ^A^γ/α ratio post transduction, with GGHI-mB-3D reaching a mean 0.35 ± 0.06 compared to 0.29 ± 0.06 seen in mock-transduced cells (*p* = 0.12, *n* = 4) and GGHI 0.31 ± 0.08 (Figure 1B, right panel, Table 3). Figure 1C shows RP-HPLC chromatograms of representative experiments, and specifically, β^S^β^S^ SCD Patient 5 and β^S^β^+^ SCD Patient 10, prior and post transduction, with GGHI-mB-3D. Transduction with the aforementioned LV led to a ^A^γ/α ratio increase from 0.16 to 0.26 in Patient 5 (Figure 1C, left panel), and from 0.31 to 0.42 in Patient 10 (Figure 1C, right panel). 

### 3.3. Increased Percentage of F-Cells following Transduction with GGHI-mB-3D Lentiviral Vector 

**Figure 2:**
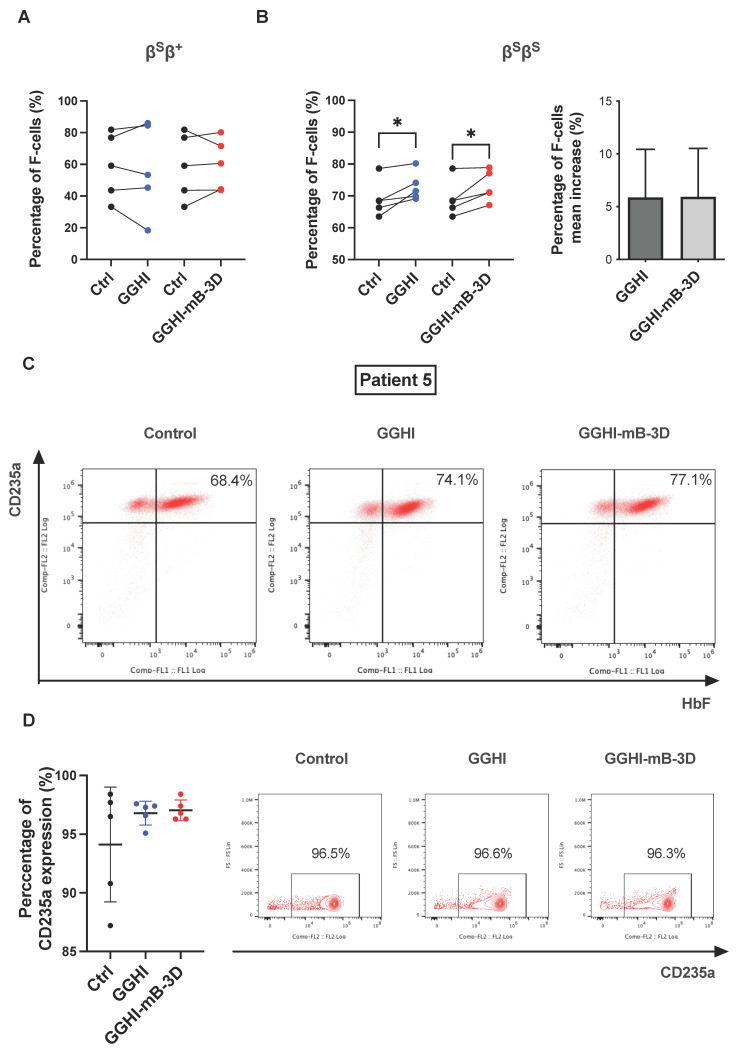
Increased production of F-cells in β^S^β^S^ SCD CD34^+^ cells: (**A**) Before-and-after plot showing F-cell percentages before and after transduction of β^S^β^+^ SCD CD34^+^ cells with GGHI (*p* = 0.746, *n* = 5, paired two-tailed *t*-test) and GGHI-mB-3D (*p* = 0.758, *n* = 5, paired two-tailed *t*-test) LVs at MOI 100. (**B**) Before-and-after plot showing F-cell percentages before and after transduction of β^S^β^S^ SCD CD34^+^ cells with GGHI (*p* = 0.038, *n* = 5, paired two-tailed *t*-test) and GGHI-mB-3D (*p* = 0.046, *n* = 5, paired two-tailed *t*-test) LVs at MOI 100 (left panel), and bar chart showing the percentage of F-cell mean increase (*p* = 0.983, *n* = 5, unpaired two-tailed *t*-test)(right panel). (**C**) Representative flow cytometry profiles showing F-cell percentages in β^S^β^S^ Patient 5. (**D**) CD235a expression at the end of in vitro differentiation (days 20–21) in the β^S^β^S^ patient cohort. Mean percentage of CD235a expression was above 90%; specifically, 94.12 ± 4.89, 96.80 ± 1.02, and 97.04 ± 0.88 in mock-transduced, GGHI-transduced, and GGHI-mB-3D-transduced samples, respectively (left panel). No statistical differences were observed between mock-transduced, and GGHI- and GGHI-mB-3D-transduced samples (*p* = 0.27 and *p* = 0.22, respectively, *n* = 5, unpaired two-tailed *t*-test). Representative flow cytometry profiles of CD235a expression in Patient 5 (right panel). Each dot corresponds to each patient. Error bars represent ±SD, * *p* ≤ 0.05.

The progression of differentiation in liquid cultures was assessed at day 20–21 by flow cytometry, using anti-HbF and anti-CD235a antibodies. Cells stained positive for both markers will be referred to as F-cells from this point onwards. Summarized results of F-cell percentages are depicted in Table 1, Table 2 and Table 3. Flow cytometry and repeated-measures ANOVA showed that transduction of only the β^S^β^S^ cohort with both GGHI and GGHI-mB-3D LVs leads to statistically significant increase in F-cell percentage (F _(2,12)_ = 5.81, *p* = 0.028). MFI did not test significantly different between samples. More specifically, and regarding F-cells in the β^S^β^+^ cohort (Figure 2A), there was no significant increase following transduction with GGHI (*p* = 0.746, *n* = 5) or GGHI-mB-3D (*p* = 0.758, *n* = 5), while in the case of β^S^β^S^ patients (Figure 2B), both vectors led to significant increase in F-cells. Transduction with either GGHI or GGHI-mB-3D led to 73 ± 4.5% and 73 ± 4.8% of F-cells, respectively (mean difference between GGHI and Ctrl of 3.9 ± 2.87, and between GGHI-mB-3D and Ctrl of 3.98 ± 3.12, Figure S3A), compared to 69 ± 5.7% observed in the control (*p* = 0.038 and *p* = 0.046, *n* = 5, respectively). Mean F-cell increase did not exceed 6% for both vectors (Figure 2B, right panel). In the representative experiment of Patient 5, GGHI led to 74.1% of F-cells and GGHI-mB-3D to 77.1%, compared to 68.4% seen in the control (Figure 2C, Table 1 and Table 2). Erythroid differentiation in β^S^β^S^ liquid cultures (days 20–21), exceeded 90% as measured by CD235a expression, demonstrating no statistical difference prior and post transduction, either with GGHI (*p* = 0.27, *n* = 5) or GGHI-mB-3D (*p* = 0.22, *n* = 5) LVs (Figure 2D (left panel) and Figure S2C). Figure 2D (right panel) shows representative percentages of CD235a expression Patient 5 at the end of differentiation.

### 3.4. Improvement in the SCD CD34^+^ Cell Phenotype In Vitro following Transduction with GGHI-mB-3D Lentiviral Vector

**Figure 3:**
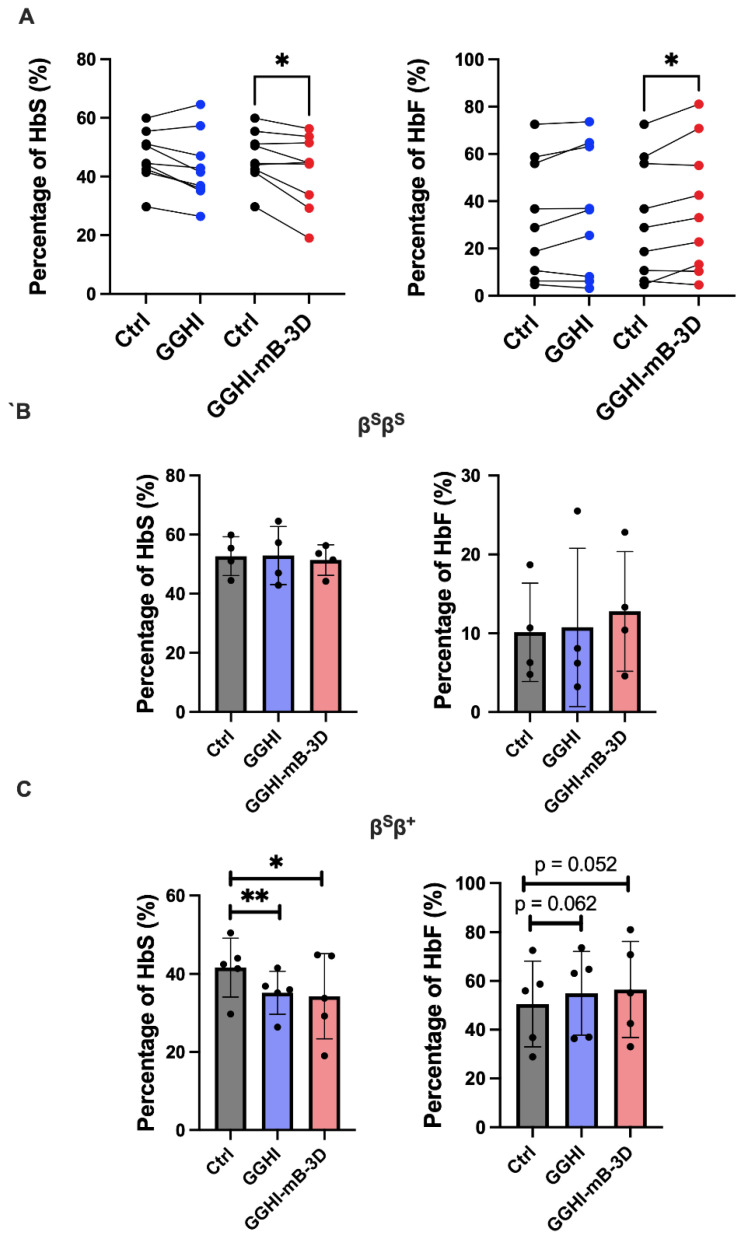
Phenotypic improvement in SCD cells in vitro following transduction with GGHI-mB-3D LV at MOI 100: (**A**) Before-and-after plots showing mean percentage of HbS (left panel) and HbF (right panel) obtained with hemoglobin (Hb) electrophoresis/CE-HPLC across all SCD patients, prior and post transduction, with GGHI (*p* = 0.053 and *p* = 0.091, respectively, *n* = 9, paired two-tailed t-test) and GGHI-mB-3D (*p* = 0.022 and *p* = 0.023, respectively, *n* = 9, paired two-tailed t-test) LVs. (**B**) Scatter plots showing mean percentage of HbS (left panel) and HbF (right panel), prior and post transduction, with GGHI (*p* = 0.91 and *p* = 0.79, respectively, *n* = 4, paired two-tailed t-test) and GGHI-mB-3D (*p* = 0.23 and *p* = 0.33, respectively, *n* = 4, paired two-tailed t-test) LVs in the β^S^β^S^ patient cohort. (**C**) Scatter plots showing mean percentage of HbS (left panel) and HbF (right panel), prior and post transduction, with GGHI (*p* = 0.005 and *p* = 0.062, respectively, *n* = 5, paired two-tailed t-test) and GGHI-mB-3D (*p* = 0.03 and *p* = 0.052, respectively, *n* = 5, paired two-tailed t-test) LVs in the β^S^β^+^ patient cohort. Each dot corresponds to each patient. Error bars represent ±SD, * *p* ≤ 0.05, ** *p* ≤ 0.01.

Due to the HU treatment received by all patients, and hence, to elevated levels in the majority of patients, reliable anti-sickling tests, as previously demonstrated by Urbinati et al. [22], could not be performed. Therefore, in order to demonstrate a potential phenotype improvement following transduction with our lentiviral vectors, we performed Hb electrophoresis/CE-HPLC, aiming to document an HbS decrease, and a concomitant HbF increase, following transduction. Repeated-measures ANOVA showed significant HbS differences between treatments (*F*_(2,24)_ = 3.74, *p* = 0.046) and across all patients. Overall, and as it can be seen in Figure 3A (left panel), transduction with GGHI-mB-3D LV only, led to a marked decrease in HbS. Specifically, the mean HbS percentage reached 41.9 ± 12.3%, compared to 46.6 ± 8.9% observed in mock-transduced cells (*p* = 0.022, *n* = 9), with a mean difference of −4.66 ± 4.94 (Figure S3A), The above decrease was followed by a concomitant HbF increase (Figure 3A, right panel), reaching 37 ± 27% compared to 33 ± 25% in mock-transduced cells (*p* = 0.023, *n* = 9), with a mean difference of 4.47 ± 4.76 (Figure S3A). Transduction with GGHI LV led to a marginal effect, with the mean HbS and HbF percentages post transduction reaching 43.1 ± 11.8% (*p* = 0.053, *n* = 9) and 35.3 ± 27% (*p* = 0.091, *n* = 9), respectively. 

Regarding the β^S^β^S^ cohort, the percentage of HbS did not test significantly different by repeated-measures ANOVA (*F*_(2,3)_ = 0.338, *p* = 0.73). Specifically, transduction with GGHI-mB-3D led to a mean HbS percentage of 51.4 ± 5.2%, compared to 52.7 ± 6.6% observed in the control (*p* = 0.23, *n* = 4), while the corresponding mean HbS percentage achieved by GGHI was 52.95 ± 9.9% (*p* = 0.91, *n* = 4) (Figure 3B, left panel and Table 2). As expected, HbF showed a small increase, without statistical significance, following GGHI and GGHI-mB-3D transduction (*p* = 0.79 and *p* = 0.33, respectively, *n* = 4) (Figure 3B, right panel). 

Interestingly, however, in the β^S^β^+^ patient cohort, both GGHI and GGHI-mB-3D LVs led to a significant reduction in HbS percentage, as demonstrated by repeated-measures ANOVA (*F*_(2,12)_ = 5.48, *p* = 0.03); particularly 35.2 ± 5.5% in the case of GGHI (*p* = 0.005, *n* = 5) (mean difference between GGHI and Ctrl of -6.42 ± 2.54, Figure S3B) and 34.3 ± 11% in the case of GGHI-mB-3D (*p* = 0.03, *n* = 5) (mean difference between GGHI-mB-3D and Ctrl of −7.33 ± 5.15, Figure S3B), compared to 41.6 ± 7.5% observed in the control (Figure 3C, left panel and Table 3). Regarding the HbF levels following transduction with GGHI and GGHI-mB-3D LVs (Figure 3C, right panel), both exhibited a marginally significant increase, reaching a mean of 55 ± 17% and 57 ± 20% (mean difference between GGHI and Ctrl of 4.40 ± 3.82, and GGHI-mB-3D and Ctrl of 5.93 ± 4.84, Figure S3B), compared to 51 ± 18% in mock-transduced cells (*p* = 0.062 and *p* = 0.052, respectively, *n* = 5).

Regarding the effects on apoptosis following vector transduction, no differences were detected between mock-transduced and transduced cells in both patient cohorts.

### 3.5. Gene Transfer Efficiency and Vector Copy Number in Transduced BFUe from SCD Patients

**Figure 4:**
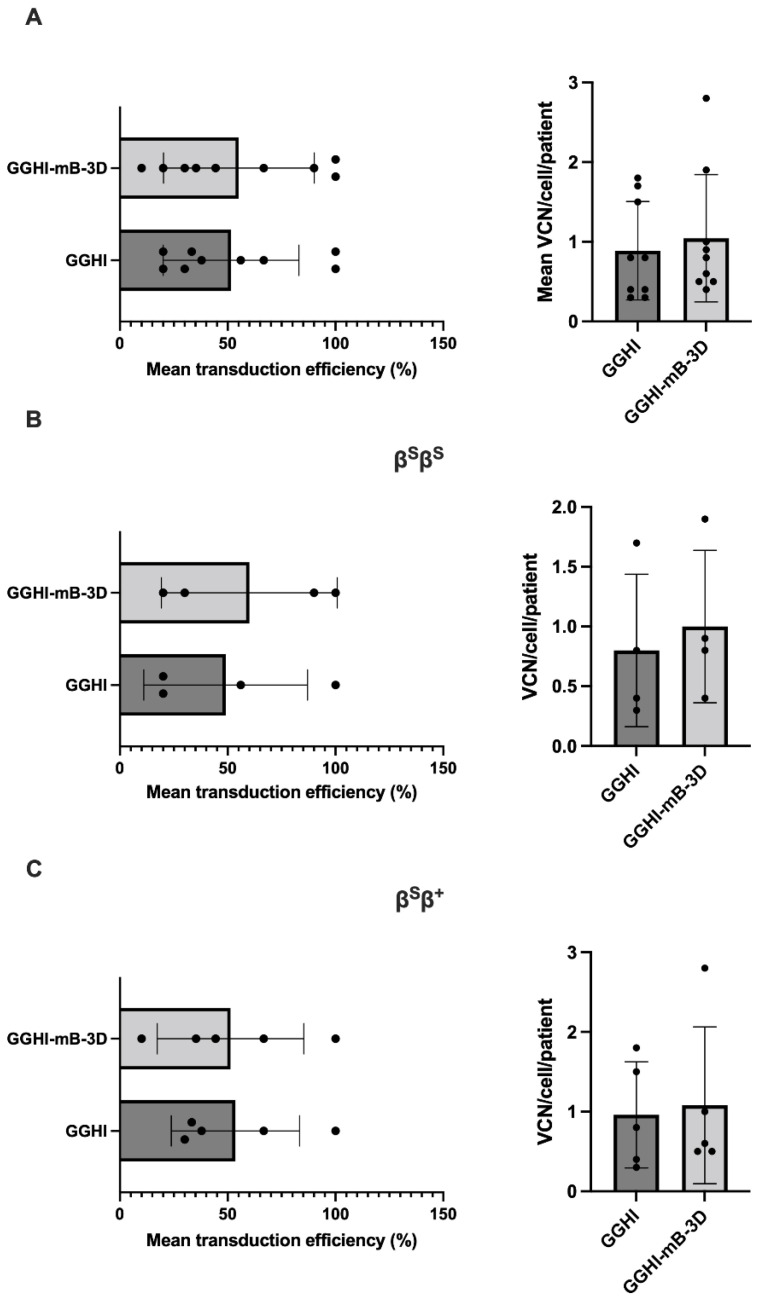
Transduction efficiency and VCN/cell demonstrated by γ-globin LVs in SCD CD34^+^ cells: (**A**) Scatter plots showing mean percentage of transduction efficiency (left) and mean VCN/cell/patient (right) across all patients and following transduction with GGHI and GGHI-mB-3D LVs at MOI 100. GGHI leads to 52 ± 31.5% and GGHI-mB-3D to 55 ± 35% transduction efficiency (*p* = 0.82, *n* = 9, unpaired two-tailed *t*-test). Mean VCN/cell/patient for GGHI and GGHI-mB-3D is 0.89 ± 0.62 and 1.04 ± 0.8, respectively (*p* = 0.65, *n* = 9, unpaired two-tailed *t*-test). (**B**) Scatter plots showing mean percentage of transduction efficiency (left) and mean VCN/cell/patient (right) in the β^S^β^S^ patient cohort and following transduction with GGHI and GGHI-mB-3D LVs at MOI 100. GGHI leads to 49 ± 38% and GGHI-mB-3D to 60 ± 40.82% transduction efficiency (*p* = 0.71, *n* = 4, unpaired two-tailed *t*-test). Mean VCN/cell/patient for GGHI and GGHI-mB-3D is 0.8 ± 0.64 and 1 ± 0.66, respectively (*p* = 0.67, *n* = 4, unpaired two-tailed *t*-test). (**C**) Scatter plots showing mean percentage of transduction efficiency (left) and mean VCN/cell/patient (right) in the β^S^β^+^ patient cohort and following transduction with GGHI and GGHI-mB-3D LVs at MOI 100. GGHI leads to 54 ± 30% and GGHI-mB-3D to 51 ± 34% transduction efficiency (*p* = 0.91, *n* = 5, unpaired two-tailed *t*-test). Mean VCN/cell/patient for GGHI and GGHI-mB-3D is 0.96 ± 0.67 and 1.1 ± 0.98, respectively (*p* = 0.83, *n* = 5, unpaired two-tailed *t*-test). Each dot corresponds to each patient. Error bars represent ±SD.

**Figure 5:**
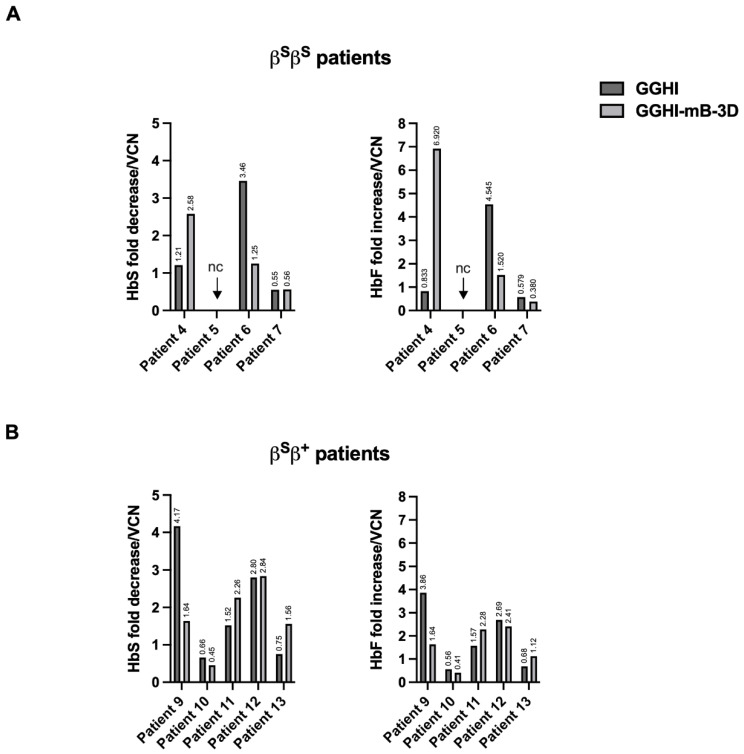
HbF and HbS fold difference in β^S^β^S^ and β^S^β^+^ SCD patients, post transduction: (**A**) Bar chart showing HbS fold decrease (left) and HbF fold increase (right) in β^S^β^S^ patients following normalization to mean VCN/cell/patient; nc, not conducted. (**B**) Bar chart showing HbS fold decrease (left) and HbF fold increase (right) in β^S^β^+^ patients following normalization to mean VCN/cell/patient.

In order to determine LV transduction efficiency, we plated CD34^+^ cells derived from both β^S^β^S^ or β^S^β^+^ patients that were either transduced with GGHI or GGHI-mB-3D or mock-transduced, in methylcellulose semi-liquid medium for colony-forming assays; 10–20 individual BFUe per patient were analyzed in each experiment by colony PCR scoring and using primers for vector-specific sequences. Gene transfer efficiency of the LVs in the CD34^+^ progenitors was determined by assessing the proportion of BFUe colonies that tested positive for vector sequences. As shown in Figure 4A (left panel) and Table 1, the mean transduction efficiencies for GGHI and GGHI-mB-3D were similar, i.e., 52 ± 32% (median 38%) and 55 ± 35% (median 44%), respectively (*p* = 0.82, *n* = 9, unpaired two-tailed *t*-test). The clonogenicities of both mock- or γ-globin-vector-transduced CD34^+^ cells were similar. Furthermore, the VCN/cell was calculated for each LV-positive BFUe from each patient, and then a mean value was extracted from all BFUe for each patient. The average VCN for GGHI-transduced cells was 0.89 ± 0.62 (median 0.8), with a range of 0.3 to 1.8, while for GGHI-mB-3D, it was 1.04 ± 0.8 (median 0.8), with a range of 0.4 to 2.8 (*p* = 0.65, *n* = 9, unpaired two-tailed *t*-test) (Figure 5A right panel and Table 1).

Transduction efficiency and VCN in the β^S^β^S^ and β^S^β^+^ patient cohorts are shown in Figure 4B,C and Table 2 and Table 3. More specifically, transduction efficiency achieved by GGHI and GGHI-mB-3D LVs in β^S^β^S^ patients was 49 ± 38% and 60 ± 41%, respectively (*p* = 0.71, *n* = 4, unpaired two-tailed *t*-test) (Figure 4B, left panel and Table 2), while mean VCN/cell was 0.8 ± 0.64 (median 0.6, with a range 0.3–1.7) for GGHI and 1 ± 0.66 (median 0.85, with a range 0.4–1.9) for GGHI-mB-3D (*p* = 0.67, *n* = 4, unpaired two-tailed *t*-test) (Figure 4B, right panel and Table 2). Transduction efficiency achieved by GGHI and GGHI-mB-3D LVs in the β^S^β^+^ cohort reached 54 ± 30% and 51 ± 34%, respectively (*p* = 0.91, *n* = 5, unpaired two-tailed *t*-test) (Figure 4C, left panel and Table 3), while mean VCN/cell was 0.96 ± 0.67 (median 0.8, with a range 0.3–1.8) and 1.1 ± 0.98 (median 0.6, with a range 0.5–2.8), respectively (*p* = 0.83, *n* = 5, unpaired two-tailed *t*-test) (Figure 4C, right panel and Table 3).

### 3.6. γ-Globin mRNA Analysis Using Quantitative Real-Time PCR 

Quantification of γ-globin production from SCD erythroid cultures showed that γ-globin mRNA is not increased significantly following transduction either with GGHI or GGHI-mB-3D lentiviral vectors at MOI 100. Specifically, we observed a mean relative fold difference of 1.03 and 0.999 (*p* = 0.75, *n* = 8, unpaired two-tailed *t*-test) in our patient cohorts, following transduction either with GGHI or GGHI-mB-3D, with the greatest increase observed in Patient 11 (Table 1 and Figure S4A). When β^S^β^S^ and β^S^β^+^ patients were analyzed separately, we failed again to detect a mean increase in γ-globin at the mRNA level, as a result of transduction with either LV, with mean relative fold difference levels in both cases to be around 1. Specifically, in β^S^β^S^ patients, the relative fold difference of γ-globin transcript was calculated 1.08 for GGHI and 0.96 for GGHI-mB-3D (*p* = 0.41, *n* = 4, unpaired two-tailed *t*-test), as shown in Table 2 and Figure S4B (left panel), while for the β^S^β^+^ patient cohort it was 0.99 and 1.04 (*p* = 0.82, *n* = 4, unpaired two-tailed *t*-test), respectively (Table 3 and Figure S4B, right panel).

## 4. Discussion

In this study, we used the previously characterized γ-globin lentiviral vector GGHI [32] and the novel optimized GGHI-mB-3D [38], both successfully assessed using thalassemic CD34^+^ cells [32,38], and investigated whether they can also improve or correct the SCD phenotype in vitro. We show that the optimized GGHI-mB-3D vector can significantly increase the ^A^γ/α ratio and HbF percentage in the SCD patient cohort, and lead to significant HbS reduction, at an average VCN of 1.0, calculated per diploid genome. This value represents the ideal target range of VCN per cell for LV-based thalassemia gene therapy [48]. Application of the Pearson’s *r* test across all patients showed very good correlation between normalised HbS fold decrease and normalized ^A^γ/α ratio (*r =* 0.9) or HbF fold increase (*r* = 0.7).

Specifically, results from RP-HPLC regarding ^A^γ/α ratio fold increase, and following normalization to VCN (Figure S5), show that four out of eight patients (Patient 5 was not included) designated as Patients 4, 8, 9, and 12 exhibited a >2-fold increase following transduction with GGHI-mB-3D. Overall, transduction with GGHI-mB-3D led to an average ^A^γ/α ratio fold increase of 1.81 ± 0.97 (*n* = 8) following normalization to VCN. The highest increase was observed in Patient 8, who led to a corrected ^A^γ/α increase of 2.96-fold. Interestingly, despite the achieved maximum ^A^γ/α ratio fold increase in Patient 8, this was not associated with a similar increase in transduction efficiency and VCN/cell (mean transduction efficiency 90% and VCN/cell 0.9). Pearson’s *r* test showed poor positive correlation between ^A^γ/α ratio fold increase and transduction efficiency (*r =* 0.16) and negative correlation between the former and VCN (*r =* −0.002). The above results suggest that expression from GGHI-mB-3D is not entirely dependent on the vector per se, but may also be influenced by the site of integration, an observation also reported by Drakopoulou et al. [38]. 

Despite the significant increase in the ^A^γ/α ratio observed with RP-HPLC, we did not detect a reciprocally lower β^S^/α ratio, following transduction with GGHI-mB-3D lentiviral vector (*p* = 0.226, *n* = 9). This may possibly be due to the restriction of the RP-HPLC analyses to the soluble populations of globin chains. To circumvent the above findings, and in order to demonstrate a potential therapeutic effect of GGHI-mB-3D and/or of GGHI, we performed hemoglobin electrophoresis or CE-HPLC analysis of lysates from mock-transduced and transduced erythroid cultures and assessed HbS and HbF expression. As expected, GGHI-mB-3D LV led to a significantly lower HbS compared with control, demonstrating a mean fold decrease of 1.643 ± 0.88 and a 2.09 ± 2.09 mean HbF fold increase, following normalization to VCN. The respective values for GGHI were 1.890 ± 1.40 and 1.92 ± 1.59.

Following patient classification according to genotype, GGHI-mB-3D failed to demonstrate a marked in vitro improvement in the β^S^β^S^ patients, suggesting higher HbF requirements for in vitro phenotypic correction in the specific patient cohort, as one would also predict from the high HbS levels before and after HU treatment in the specific patient cohort (Figure S2A,B and Table 2 and Table 3). Mean HbS fold decrease following normalization to VCN in the specific cohort was 1.46 ± 1.03 (*n = 3*), with the maximum value observed in Patient 4 who exhibited a 2.58-fold HbS decrease (Figure 5A, left panel), demonstrating also the maximum, i.e., 6.92-fold HbF increase (Figure 5A, right panel). On the contrary, transduction of β^S^β^+^ cells with GGHI-mB-3D led to a 1.75 ± 0.89 (*n* = 5) mean HbS fold decrease and 1.57 ± 0.83 (*n = 5*) mean HbF fold increase, following normalization to VCN. Out of five β^S^β^+^ patients, transduction with GGHI-mB-3D led to HbS decrease in four patients, with the highest value observed in Patient 12, who exhibited a HbS decrease of 2.84-fold (Figure 5B, left panel), also showing the highest, i.e., 2.41-fold, HbF increase (Figure 5B, right panel). Pearson’s *r* test showed moderate positive correlation between transduction efficiency and normalized HbS fold decrease (*r* = 0.56) and normalized HbF fold increase (*r =* 0.59).

Regarding the GGHI vector in the β^S^β^S^ patient cohort, the mean HbS fold decrease following normalization to VCN reached 1.74 ± 1.53, showing no statistical significance. The most profound effect was demonstrated by Patient 6, who demonstrated a 3.46-fold HbS decrease (Figure 5A, left panel), also showing the highest, i.e., 4.55-fold, HbF increase (Figure 5A, right panel). On the contrary, and in the case of β^S^β^+^ patients, the mean HbS fold decrease and HbF fold increase were 1.98 ± 1.5 and 1.87 ± 1.4, respectively, *(n =* 5), both following normalization to VCN. Pearson’s *r* test showed no positive correlation between transduction efficiency and normalized HbF fold increase (*r* = −0.03) or HbS fold decrease (*r* = −0.09). 

With regards to the γ-globin mRNA levels (Figure S4), despite the marked HbF increase observed following transduction with GGHI, and, most importantly, with GGHI-mB-3D γ-globin LVs, we failed to demonstrate a marked mean increase in γ-globin transcripts relative to α-globin. The latter is in contrast with Urbinati et al. [22], in SCD cells, who showed that the γ-globin lentiviral vector V5m3-400 managed to increase γ-globin transcripts by more than 7-fold. However, this finding was based on a rather small number of three patients, while in our series we investigated a more representative cohort of eight informative patients (Patients 7 and 9 were not included due to limited cell number). We also managed to detect a small increase in at least three patients; specifically, Patients 6, 8, and 11, who showed the highest increase following transduction with GGHI, and Patients 6, 10, and 11, who showed increased γ-globin transcripts following transduction with GGHI-mB-3D LV. Most patients with increased γ-globin mRNA levels, displayed a concomitant HbF increase. A possible explanation for not detecting a similar high γ-globin increase at the mRNA level, as Urbinati et al. noted, could be attributed to the late sampling for RNA isolation. Compared to Urbinati et al. [22], we carried out RNA isolation 1 week later during erythroid differentiation, i.e., at day 20–21 instead of day 14, which may have resulted in the underestimation of expression of vector-derived γ-globin mRNA. As documented elsewhere [49,50], in vitro erythroid differentiation partially recapitulates ontogenesis and hemoglobin switching for ES-derived [49], as well as for adult peripheral blood CD34^+^-derived progenitors [50], with a shift to the contribution of embryonic to fetal and then to adult globin chains on the background of an overall increasing globin expression during erythroid differentiation. Since both the vector-derived ^A^γ gene and the endogenous one share identical cis-acting sequences, and thus undergo the same transcriptional regulation, it is conceivable that an endogenous fetal to adult switch from γ-globin to β-globin and/or β^S^ at late stages of erythroid differentiation would result in relatively lower γ-globin mRNA levels and a higher overall β^S^ contribution. 

Regarding the differences between the GGHI and GGHI-mB-3D performance, despite the significant ^A^γ/α ratio and HbF increase, as well as the HbS decrease demonstrated only by GGHI-mB-3D LV compared to mock-transduced cells, we did not observe significant differences between the former and GGHI, possibly due to extensive variation among patient samples, an observation also documented in a previous study by Drakopoulou et al. [38].

In line with our previous observations in thalassemia patients, and of others in SCD patients [22], the mean VCN calculated for GGHI and GGHI-mB-3D lentiviral vectors was around 1.0 (*p* = 0.65, *n* = 9, unpaired two-tailed *t*-test), reflecting a near-ideal value for a clinical setting and a potentially therapeutic outcome, as reported by Kanter et al. [51]. VCN achieved among the β^S^β^S^ and β^S^β^+^ patient cohorts did not test significantly different between GGHI (*p* = 0.51, *n* = 4, unpaired two-tailed *t*-test) and GGHI-mB-3D (*p* = 0.66, *n* = 4, unpaired two-tailed *t*-test) LVs. With regards to transduction efficiency, GGHI and GGHI-mB-3D both achieved an overall gene transfer of 52% and 55%, respectively (*p* = 0.82, *n* = 9, unpaired two-tailed *t*-test). Again, no significant differences were observed in transduction efficiency among the β^S^β^S^ and β^S^β^+^ cohorts between GGHI (*p* = 0.75, *n* = 4, unpaired two-tailed *t*-test) and GGHI-mB-3D (*p* = 0.74, *n* = 5, unpaired two-tailed *t*-test) LVs.

It should be emphasized that our vectors, harboring several erythroid-specific regulatory elements, to the best of our knowledge, are the only LCR-free, SIN, and insulated globin vectors that can be employed to efficiently transduce erythroid progenitor cells and successfully drive the human γ-globin gene to nearly therapeutic levels in both thalassemic [32,38] and SCD CD34^+^ cells, as shown in this study. In view of the ongoing clinical trials for thalassemia and SCD, one of the main strengths of these vectors is their safety feature, due to their insulation and to the lack of any LCR regulatory elements, which have been recently shown to be active in early hematopoietic progenitor cells [52], and thus are capable of trans-activating cancer-related genes, with all the known associated risks for the patient [36,37,52,53].

In conclusion, despite the significant and potentially therapeutic HbF increase observed in SCD patients with the optimized γ-globin lentiviral vector GGHI-mB-3D, a major limitation of our strategy, primarily regarding the precise assessment and comparison of the HbF increase in each patient and among the β^S^β^S^ and β^S^β^+^ cohorts, is the established inherent genetic and epigenetic heterogeneity per se of these patients, coupled with the lack of information regarding important HbF genetic modifiers, such *XmnI, HBS1L-MYB*, and *BCL11A* [54], in the two cohorts. These major SNPs associated with high HbF levels, account for more than 20% of the HbF level variations among SCD patients [55], and affect both the severity and the therapeutic outcome in SCD, including patients under HU treatment [56]. In a recent systematic review by Sales et al. [57], the authors concluded that genetic variations in multiple loci, such as SNPs located at intron 2 of the *BCL11A* gene, can affect both baseline HbF and HbS levels in response to HU treatment in patients with SCD. Therefore, information on these SNPs, together with larger patient cohorts of different ethnic origin, would significantly contribute to a more effective assessment of the GGHI-mB-3D γ-globin vector in the context of sickle cell disease gene therapy [58].

## 5. Conclusions

In summary, we show that the optimized LCR-free GGHI-mB-3D lentiviral vector, which carries novel regulatory elements, can significantly increase the ^A^γ/α ratio and HbF in CD34^+^ cells from SCD patients, an increase accompanied by a concomitant HbS decrease, demonstrating a potentially therapeutic outcome in vitro. These data suggest that despite the non-significant differences between the optimized GGHI-mB-3D and GGHI lentiviral vectors, the former demonstrates an increased potential of improving the SCD phenotype in vitro, and thus, can eventually provide a significant therapeutic benefit in the context of future clinical trials for patients with sickle cell disease. 

## Figures and Tables

**Table 1:** Detailed results from all patients.

Sample	Genotype	Mutation	Source	F-Cells (FACS)			^A^γ/α Ratio (RP-HPLC)			HbS Decrease (%)		TSD Efficiency (%)		Mean VCN/Cell		Relative Fold Difference of γ-mRNA Transcripts	
				Ctrl	GGHI	GGHI-mB-3D	Ctrl	GGHI	GGHI-mB-3D	GGHI	GGHI- mB-3D	GGHI	GGHI- mB-3D	GGHI	GGHI- mB-3D	GGHI	GGHI- mB-3D
#4	β^S^β^S^	HbS/HbS	PB	66.3	69.1	71.1	0.21	0.11	0.24	0	3.3	20	20	0.8	0.4	1.04	1
#5	β^S^β^S^	HbS/HbS	PB	68.4	74.1	77.1	0.16	0.46	0.26	8	0	-	-	-	-	0.93	0.76
#6	β^S^β^S^	HbS/HbS	PB	63.5	71.5	67.1	0.25	0.24	0.23	3.6	0.7	20	30	0.3	0.8	1.15	1.28
#7	β^S^β^S^	HbS/HbS	PB	78.6	80.2	78.9	0.17	0.15	0.19	0	6	100	100	1.7	1.9	-	-
#8	β^S^β^S^	HbS/HbS	PB	68.5	69.9	71	0.09	0.25	0.24	-	-	56	90	0.4	0.9	1.19	0.79
#9	β^S^β^+^	HbS/ IVS1-110	PB	81.9	84.5	71.5	0.21	0.23	0.29	20	0	30	10	0.3	0.6	-	-
#10	β^S^β^+^	HbS/ IVS1-110	PB	59.1	53.4	60.6	0.31	0.31	0.42	15.3	20.5	33.3	35.3	1.8	2.8	1.06	1.09
#11	β^S^β^+^	HbS/ IVS1-110	PB	43.6	45.3	43.8	0.30	0.28	0.30	17.8	11.7	100	100	0.8	0.5	1.29	1.34
#12	β^S^β^+^	HbS/ IVS1-1	PB	33.2	18.4	44.3	0.35	0.42	0.38	10.9	29.5	66.7	66.7	0.4	0.5	0.56	0.78
#13	β^S^β^+^	HbS/ IVS1-110	PB	76.9	86	80.2	-	-	-	11.1	36	37.9	44.4	1.5	1.0	1.06	0.95
Average				64	65	67	0.23	0.27	0.28	9.6	12	52	55	0.89	1.04	1.03	0.999
*p*-value					0.59	0.19		0.30	0.02	0.66		0.82		0.65		0.75	

Note: Dashes indicate that the relevant assays were not conducted due to limited number of cells; PB, peripheral blood; TSD, transduction; VCN, vector copy number; RP-HPLC, reversed-phase HPLC.

**Table 2:** Detailed results from β^S^β^S^ patients.

Sample	Genotype	Mutation	F-Cells (FACS)			^A^γ/α Ratio (RP-HPLC)			HbS (%)			HbF (%)			TSD Efficiency (%)		Mean VCN/cell		Relative Fold Difference of γ-mRNA Transcripts	
			Ctrl	GGHI	GGHI-mB-3D	Ctrl	GGHI	GGHI- mB-3D	Ctrl	GGHI	GGHI- mB-3D	Ctrl	GGHI	GGHI- mB-3D	GGHI	GGHI- mB-3D	GGHI	GGHI- mB-3D	GGHI	GGHI- mB-3D
#4	β^S^β^S^	HbS/HbS	66.3	69.1	71.1	0.21	0.11	0.24	55.4	57.3	53.6	4.8	3.2	13.3	20	20	0.8	0.4	1.04	1
#5	β^S^β^S^	HbS/HbS	68.4	74.1	77.1	0.16	0.46	0.26	51.1	47	51.5	10.7	8.1	10.4	-	-	-	-	0.93	0.76
#6	β^S^β^S^	HbS/HbS	63.5	71.5	67.1	0.25	0.24	0.23	44.5	42.9	44.2	18.7	25.5	22.8	20	30	0.3	0.8	1.15	1.28
#7	β^S^β^S^	HbS/HbS	78.6	80.2	78.9	0.17	0.15	0.19	59.9	64.6	56.3	6.3	6.2	4.6	100	100	1.7	1.9	-	-
#8	β^S^β^S^	HbS/HbS	68.5	69.9	71	0.09	0.25	0.24	-	-	-	-	-	-	56	90	0.4	0.9	1.19	0.79
Average			69	73	73	0.18	0.24	0.23	52.7	53	51.4	10.13	10.75	12.78	49	60	0.8	1	1.08	0.96
*p*-value				0.038	0.046		0.41	0.14		0.91	0.23		0.79	0.33	0.71		0.67		0.41	

Note: Dashes indicate that the relevant assays were not conducted due to limited number of cells; PB, peripheral blood; TSD, transduction; VCN, vector copy number; RP-HPLC, reversed-phase HPLC.

**Table 3:** Detailed results from β^S^β^+^ patients.

Sample	Genotype	Mutation	F-Cells (FACS)			^A^γ/α Ratio (RP-HPLC)			HbS (%)			HbF (%)			TSD Efficiency (%)		Mean VCN/Cell		Relative Fold Difference of γ-mRNA Transcripts	
			Ctrl	GGHI	GGHI-mB-3D	Ctrl	GGHI	GGHI-mB-3D	Ctrl	GGHI	GGHI-mB-3D	Ctrl	GGHI	GGHI- mB-3D	GGHI	GGHI- mB-3D	GGHI	GGHI- mB-3D	GGHI	GGHI- mB-3D
#9	β^S^β^+^	HbS/ IVS1-110	81.9	84.5	71.5	0.21	0.23	0.29	44.01	35.19	44.86	55.97	64.81	55.14	30	10	0.3	0.6	-	-
#10	β^S^β^+^	HbS/ IVS1-110	59.1	53.4	60.6	0.31	0.31	0.42	42.45	35.96	33.77	36.77	36.92	42.53	33.3	35.3	1.8	2.8	1.06	1.09
#11	β^S^β^+^	HbS/ IVS1-110	43.6	45.3	43.8	0.30	0.28	0.30	50.50	41.50	44.60	28.90	36.40	33.00	100	100	0.8	0.5	1.29	1.34
#12	β^S^β^+^	HbS/ IVS1-1	33.2	18.4	44.3	0.35	0.42	0.38	41.40	36.90	29.20	58.70	63.10	70.80	66.7	66.7	0.4	0.5	0.56	0.78
#13	β^S^β^+^	HbS/ IVS1-110	76.9	86	80.2	-	-	-	29.70	26.40	19.00	72.50	73.60	81.00	37.9	44.4	1.5	1.0	1.06	0.95
Average			59	58	60	0.29	0.31	0.35	41.61	35.19	34.29	50.57	54.97	56.49	54	51	0.96	1.1	0.99	1.04
*p*-value				0.75	0.76		0.45	0.12		0.005	0.03		0.062	0.052	0.91		0.83		0.82	

Note: Dashes indicate that the relevant assays were not conducted due to limited number of cells; PB, peripheral blood; TSD, transduction; VCN, vector copy number; RP-HPLC, reversed-phase HPLC.

## Data Availability

The data that support the findings of this study are available on request from the first author.

## Supplementary Materials

The following supporting information can be downloaded at: https://www.mdpi.com/article/10.3390/v14122716/s1. Figure S1: (**A**) Schematic representation of the experimental procedure. (**B**) Table showing corresponding titers (TU/mL) of different virus batches (LOTs) for GGHI (*n* = 6) and GGHI-mB-3D (*n* = 5) γ-globin LVs (left panel), and bar chart showing mean GGHI and GGHI-mB-3D titers (*p* = 0.902, unpaired two-tailed *t*-test) (right panel). Figure S2: (**A**) Baseline HbF and HbS expression prior to hydroxyurea (HU) treatment in the β^S^β^S^ (*n* = 5) (upper panel), and β^S^β^+^ patient cohort (*n* = 5) (lower panel). (**B**) HbS expression prior and post HU treatment in β^S^β^S^ and β^S^β^+^ patients. (**C**) CD235a expression at the end of the in vitro differentiation. Figure S3: Estimation plots for all comparisons with statistical significance. Figure S4: Performance of γ-globin LVs at the RNA level: (**A**) Bar chart showing relative fold difference of γ-mRNA transcripts in different patients (*n* = 8). (**B**) Bar charts showing relative fold difference of γ-mRNA transcripts in the β^S^β^S^ patient cohort (*n* = 4) (left panel) and in the β^S^β^+^ patient cohort (*n* = 4) (right panel). Figure S5: ^A^γ/α ratio fold increase per patient post transduction with GGHI and GGHI-mB-3D LVs and following normalization to VCN.
