# Supplementary material for: The Optimized γ-Globin Lentiviral Vector GGHI-mB-3D Leads to Nearly Therapeutic HbF Levels In Vitro in CD34+ Cells from Sickle Cell Disease Patients
Source: Viruses. 2022 Dec 5;14(12):2716. doi: 10.3390/v14122716 (PMC9783242; doi:10.3390/v14122716)
Supplement: Supplementary file 1 [file viruses-14-02716-s001.zip › viruses-1953319-supplementary.pdf]

viruses

Supplementary Figures

A

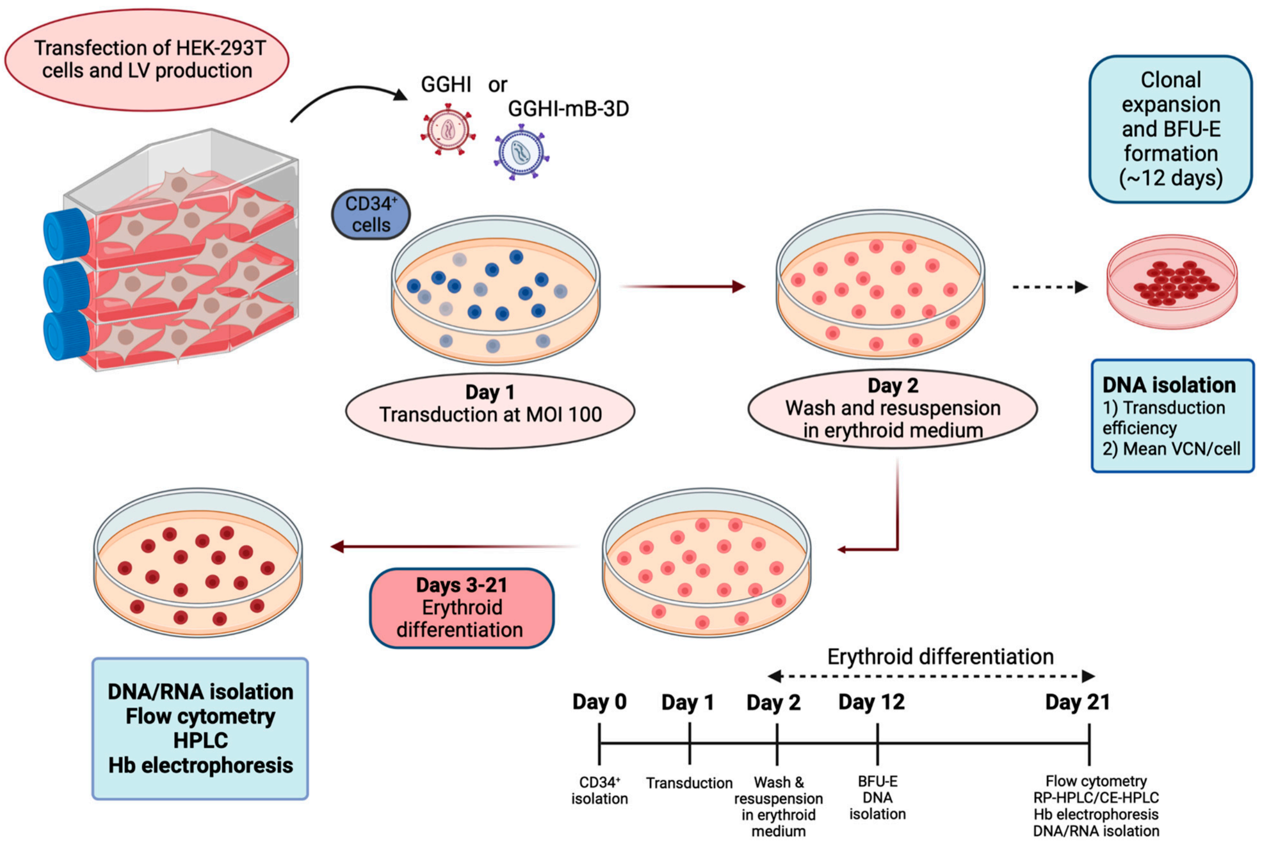

B

	GGHI	GGHI-mB-3D
LOT 1	3.7e+008	6.95e+007
LOT 2	1.0e+008	2.60e+008
LOT 3	2.2e+008	1.20e+008
LOT 4	1.2e+008	1.60e+008
LOT 5	1.1e+008	1.70e+008
LOT 6	6.0e+007	

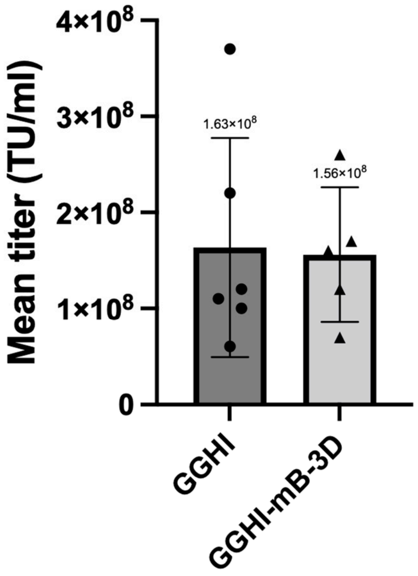

Figure S1. (A) Schematic representation of the experimental procedure, created with Biorender.com (accessed on 11 November 2022) **(B)** Table showing corresponding titers (TU/mL) of different virus batches (LOTs) for GGHI ($n = 6$) and GGHI-mB-3D ($n = 5$) γ-globin lentiviral vectors (left panel) and bar chart showing mean GGHI and GGHI-mB-3D titers ($p = 0.902$, unpaired two-tailed *t-test*) (right panel). Error bars represent ±SD.

A

$\beta^S\beta^S$

	HbF	HbS
Patient 4	5.4	87.3
Patient 5	7.9	83.7
Patient 6	2.0	91.4
Patient 7	9.2	85.4
Patient 8	6.9	85.7

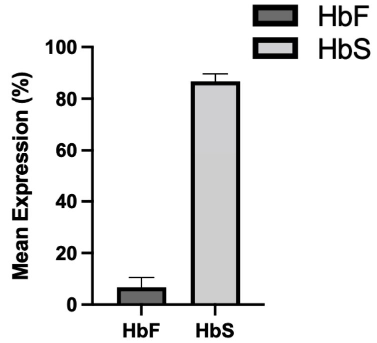

$\beta^S\beta^+$

	HbF	HbS
Patient 9	4.5	79.6
Patient 10	5.5	81.2
Patient 11	4.6	80.0
Patient 12	5.3	75.2
Patient 13	16.0	69.6

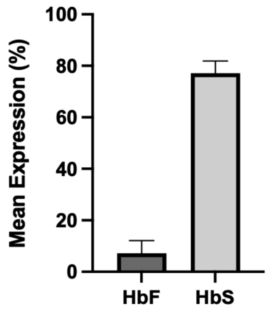

B

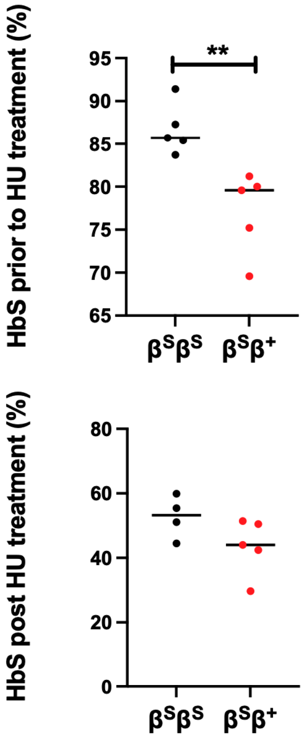

C

$\beta^S\beta^S$

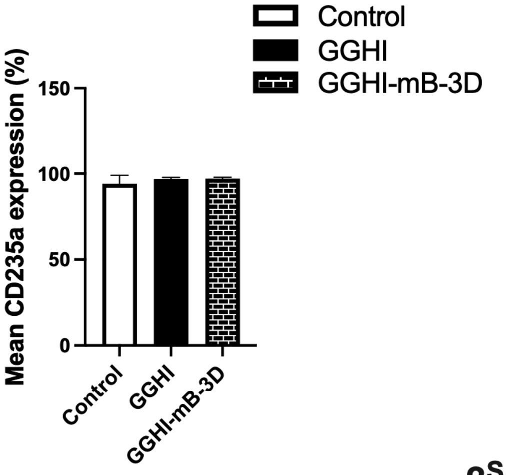

	Control	GGHI	GGHI-mB-3D
Patient 4	97.7	97.3	98.4
Patient 5	96.5	96.6	96.3
Patient 6	87.2	97.4	96.8
Patient 7	98.4	97.6	97.4
Patient 8	90.8	95.1	96.3

$\beta^S\beta^+$

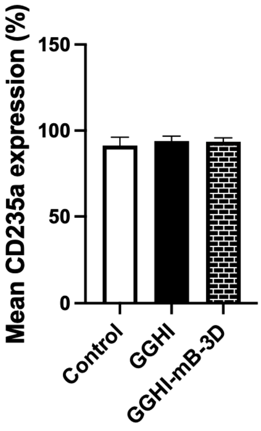

	Control	GGHI	GGHI-mB-3D
Patient 9	98.2	98.5	97.3
Patient 10	91.6	91.8	90.9
Patient 11	93.4	93.3	92.9
Patient 12	87.2	91.2	92.9
Patient 13	85.9	94.7	93.6

Figure S2. (A) Baseline HbF and HbS expression prior to hydroxyurea (HU) treatment. The mean percentage of HbF and HbS in $\beta^S\beta^S$ patient cohort was 6.28 ± 2.77% and 86.7 ± 2.92% (*n* = 5) respectively (upper panel), while for $\beta^S\beta^+$ patient cohort it reached 7.18 ± 4.95% and 77.12 ± 4.78% (*n* = 5) respectively (lower panel) **(B)** HbS expression prior (upper panel) and post (lower panel) HU treatment in $\beta^S\beta^S$ and $\beta^S\beta^+$ patients· The former cohort expressed significantly higher percentage of HbS compared to β^S/β^+ prior to hydroxyurea treatment ($p = 0.0051$, $n = 5$). There was no statistical difference between cohorts post HU treatment ($p = 0.1274$, $\beta^S\beta^S$: $n = 4$, $\beta^S\beta^+$: $n = 5$) **(C)** CD235a expression at the end of *in vitro* differentiation (D19-D20) in $\beta^S\beta^S$ patients (upper panel) and $\beta^S\beta^+$ patients (lower panel). Each dot corresponds to each patient. Error bars represent ±SD.

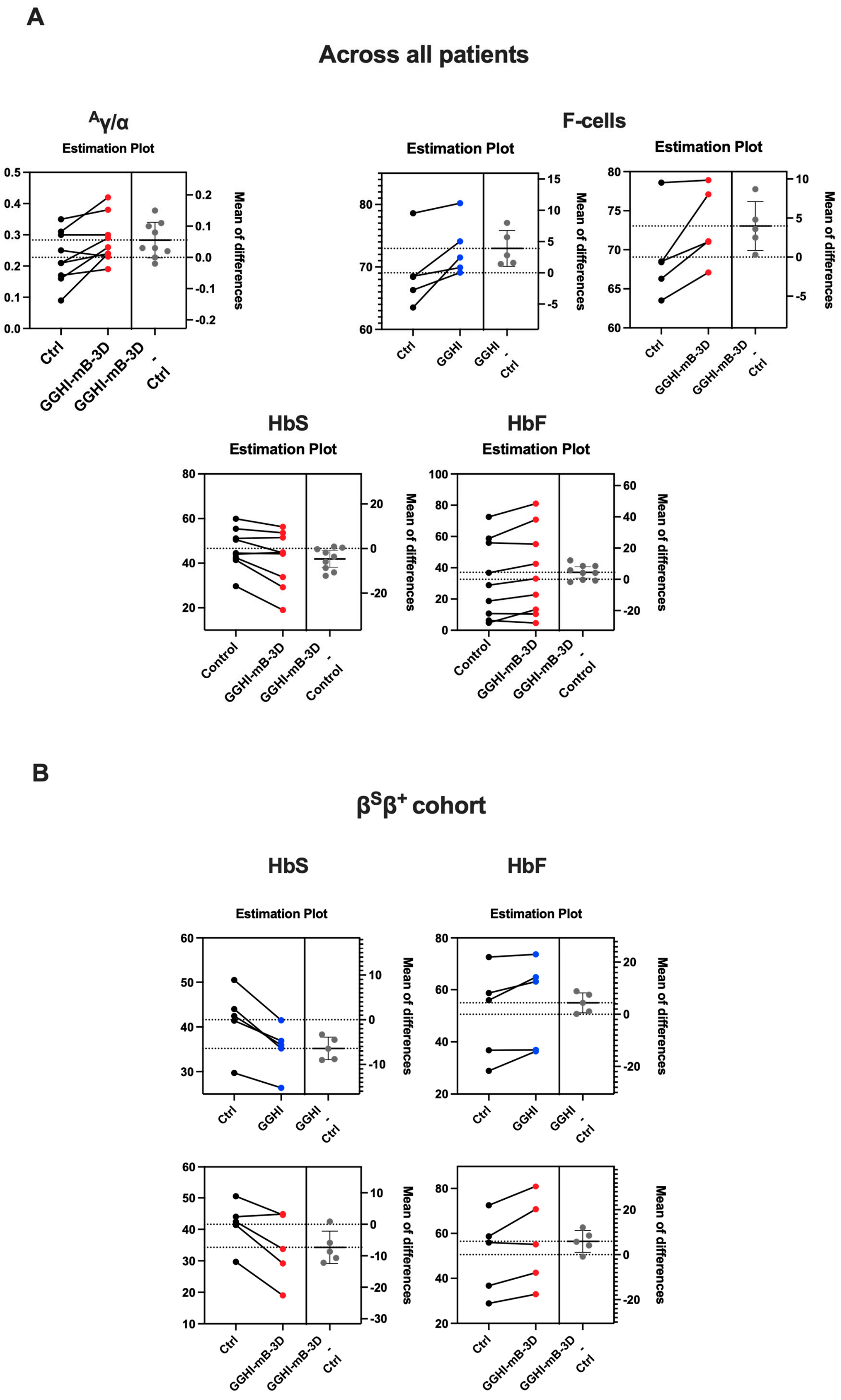
A
Across all patients
Aγ/α
Estimation Plot
Mean of differences
Ctrl
GGHI-mB-3D
GGHI-mB-3D - Ctrl
F-cells
Estimation Plot
Ctrl
GGHI
GGHI - Ctrl
HbS
Estimation Plot
Control
GGHI-mB-3D
GGHI-mB-3D - Control
HbF
B
βSβ+ cohort
HbS
HbF

Figure S3. Esimation plots for all comparisons with statistical significance. Each dot corresponds to each patient.

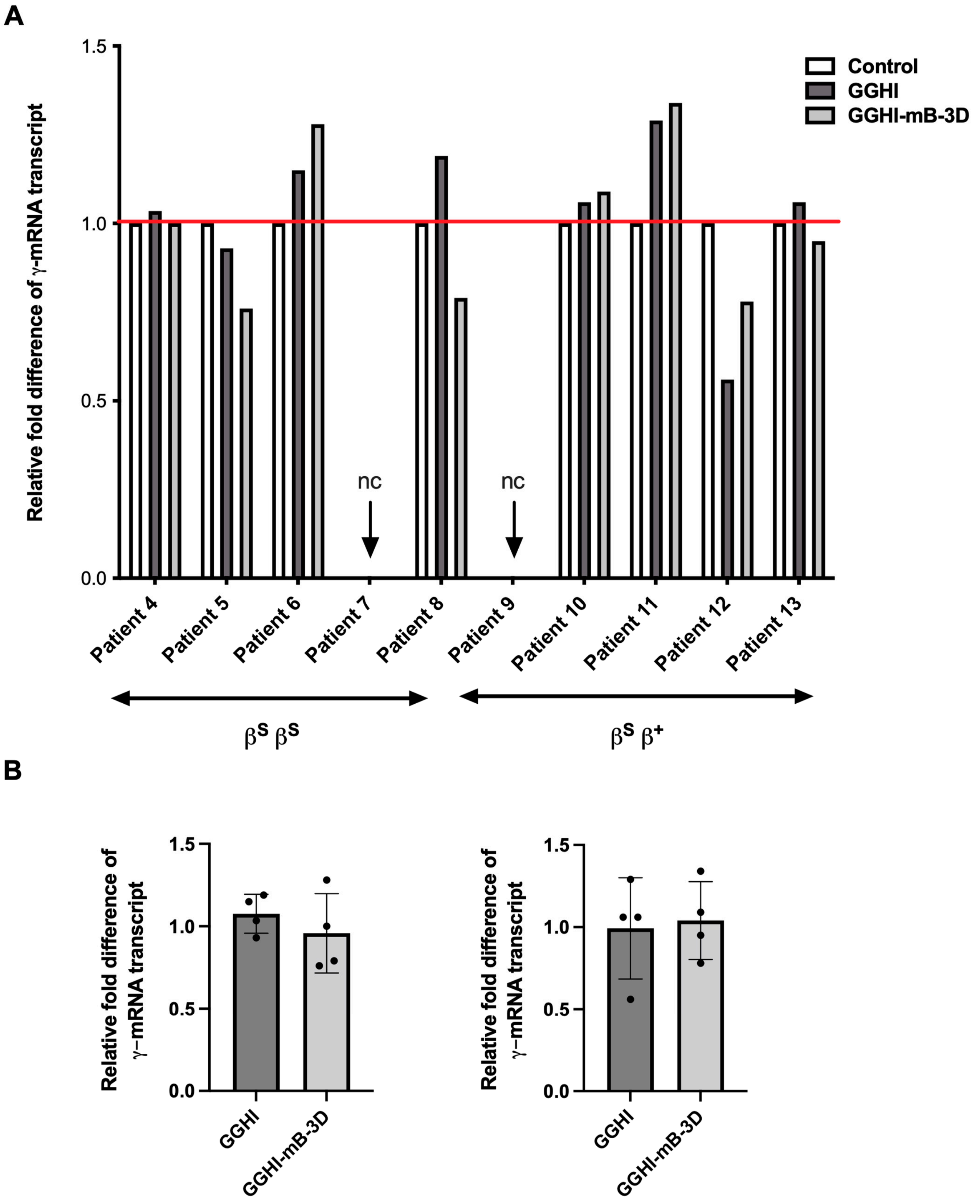

Figure S4. Performance of γ-globin LVs at the RNA level **(A)** Bar chart showing relative fold difference of γ-mRNA transcripts in different patients (*n* = 8) **(B)** Bar charts showing relative fold difference of γ-mRNA transcripts in the $\beta^S\beta^S$ patient cohort (*n* = 4) (left

panel) and in the $\beta^S\beta^+$ patient cohort (n = 4) (right panel). Normalization was carried out to α-globin gene. Each dot corresponds to each patient. Error bars represent ±SD.

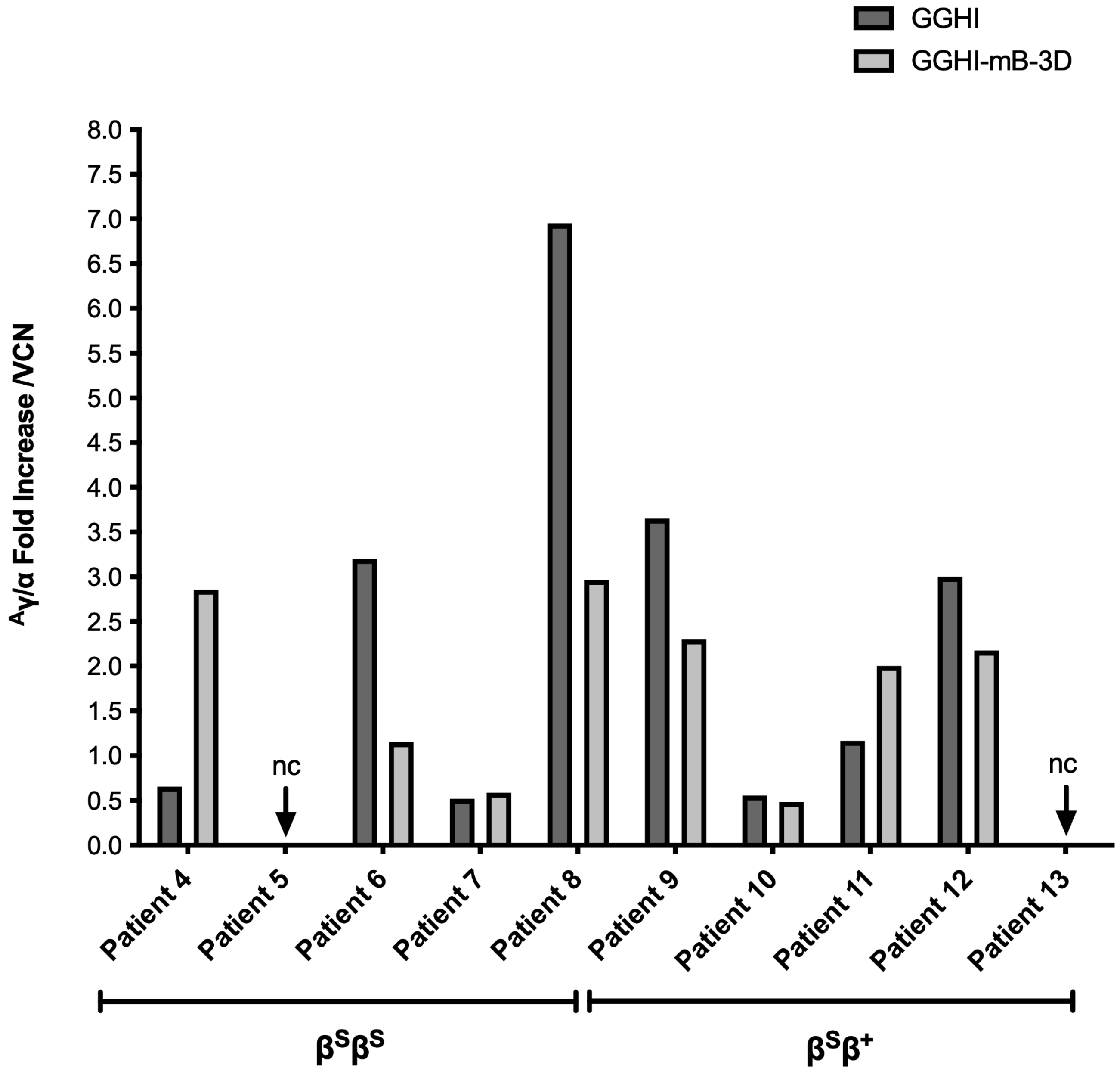

Figure S5. $^A\gamma/\alpha$ ratio fold increase per patient post-transduction with GGHI and GGHI-mB-3D lentiviral vectors and following normalization to mean VCN/cell. nc: not conducted due to limited number of cells.
